# Silver diamine fluoride versus therapeutic sealants for the arrest and prevention of dental caries in low-income minority children: study protocol for a cluster randomized controlled trial

**DOI:** 10.1186/s13063-018-2891-1

**Published:** 2018-09-26

**Authors:** Ryan Richard Ruff, Richard Niederman

**Affiliations:** 1 Department of Epidemiology and Health Promotion, New York University College of Dentistry, 433 First Avenue, Room 712, New York, NY 10010 USA; 2 New York University College of Global Public Health, New York, NY USA

**Keywords:** Dental caries, Caries arrest, Caries prevention, Quality of life, Education, Silver diamine fluoride, Sealants, Interim therapeutic restorations

## Abstract

**Background:**

Dental caries is the most prominent childhood disease in the world. In the United States, more than 50% of children have experienced caries. Untreated caries can have negative impacts on quality of life, academic performance, and school attendance. To reduce oral health disparities, multiple organizations recommend school-based caries prevention.

**Methods/design:**

A longitudinal, cluster randomized, non-inferiority trial will be conducted in low-income children from primarily Hispanic/Latino backgrounds currently enrolled in public elementary schools in New York City, New York, United States, from 2018 to 2023. The primary objective is to compare the non-inferiority of silver diamine fluoride and fluoride varnish versus glass ionomer therapeutic sealants and fluoride varnish in the arrest and prevention of dental caries. Secondary objectives are to evaluate differences in effectiveness when care is provided by nurses versus dental hygienists and assess the impact of prevention on oral health-related quality of life and educational outcomes. Caries arrest will be evaluated after 2 years, and caries prevention and secondary outcomes will be assessed at the completion of the study. Data analysis will follow intent-to-treat, and statistical analyses will be conducted using a two-sided significance level of 0.05.

**Discussion:**

The comparative effectiveness of alternative caries prevention delivery models is considered to be one of the highest research priorities in the United States. Many treatments are currently available to prevent and arrest dental caries. The simplicity and affordability of silver diamine fluoride may be a viable alternative for the prevention of dental caries in high-risk children.

**Trial registration:**

U.S. National Library of Medicine, www.clinicaltrials.gov, ID: NCT03442309. Registered on 22 February 2018.

**Electronic supplementary material:**

The online version of this article (10.1186/s13063-018-2891-1) contains supplementary material, which is available to authorized users.

## Background

Dental caries (tooth decay) is a natural process in which bacteria in the biofilm cause fluctuations in pH that can lead to an erosion of dental hard tissues and result in visible lesions [1], and is the most prevalent childhood disease in the world [2, 3]. Untreated dental caries affects more than 20% of elementary school-aged children in the United States, and over 50% of children have ever experienced caries. For low-income and minority-group children, caries experience can exceed 70% and the prevalence of untreated caries is greater than 30% [4–6]. Persistent untreated dental caries in children can lead to pulpal involvement and abscess, and contributes to oral pain [7, 8]. In 2017, a comprehensive review of dental caries concluded that though the overall prevalence of caries has decreased worldwide, the burden of disease is still significant across all age groups and is most prominent among low-income populations [9].

Poor oral health can have negative long-term impacts on quality of life, school attendance, and academic performance. Research has consistently showed that caries experience negatively affects oral health-related quality of life across multiple age groups, socioeconomic levels, and children from different countries [10–12]. Further, children with dental caries and associated toothache have been shown to have increased absenteeism and reduced academic performance [13–19]. Notably, children with caries who received dental care had significant improvement in their quality of life [20].

Low-income and minority-group children face profound oral health inequities. Lower dental service utilization is found among low-income and minority-group families, and the bulk of services received focused on treating existing issues while neglecting preventive care [21]. For example, sealant use is lowest in low-income children and few of these children reported visiting a dentist in the previous year [6, 22, 23]. Typically, it is children most at risk of oral diseases who lack access to preventive services [24], and many traditional office-based dentists do not provide preventive care [25]. To address this unmet need, multiple organizations and institutions recommend school-based caries prevention programs as a supplement to traditional care in order to increase access to dental services and reduce oral health inequities [24, 26–28]. Among the many treatments available to arrest and/or prevent dental caries, leading options include water fluoridation, fluoride toothpaste, fluoride varnish, sealants and/or interim therapeutic restorations (ITR), and silver diamine fluoride (SDF), each of which has been shown to have varying levels of efficacy in clinical trials [29–34].

The Institute of Medicine considers the study of caries-prevention models to be a “high priority” topic in comparative effectiveness research [35]. In addition to the potential differences in mode of delivery (e.g., mobile dental vans versus school-based oral health clinics), the impact of alternative primary and/or secondary caries prevention agents when used in pragmatic settings is largely unknown. Additionally, there is a noticeable lack of research on the potential impact of caries prevention on subsequent quality of life and educational performance. We present a longitudinal, cluster randomized, non-inferiority trial designed to compare two packages of treatments with both primary and secondary caries prevention agents: a “simple” prevention package consisting of fluoride varnish and SDF and a “complex” package consisting of fluoride varnish, sealants, and ITR. The primary outcomes of the trial are caries arrest and caries prevention. Secondary outcomes include quality of life, academic performance, and school absences. Additionally, the comparative effectiveness of simple prevention when provided by dental hygienists versus school nurses will be assessed. The primary population is low-income Hispanic/Latino children who attend elementary schools in an urban school district, known to be one of the highest-need oral health populations in the region. It is hypothesized that simple caries prevention is non-inferior to complex care.

## Methods/design

This is a longitudinal, pragmatic, cluster randomized, non-inferiority clinical trial consisting of SDF combined with fluoride varnish versus therapeutic sealants with fluoride varnish given biannually to each study participant in primary schools. This trial is reported following the Standard Protocol Items: Recommendations for Interventional Trials (SPIRIT) guidelines and has received approval from the New York University (NYU) School of Medicine Institutional Review Board (IRB) (#i17–00578). Any changes to the study protocol will be communicated to the IRB and funder in quarterly reports, and investigators will cooperate with any independent audit on behalf of the IRB or funding organization.

**Fig. 1:**
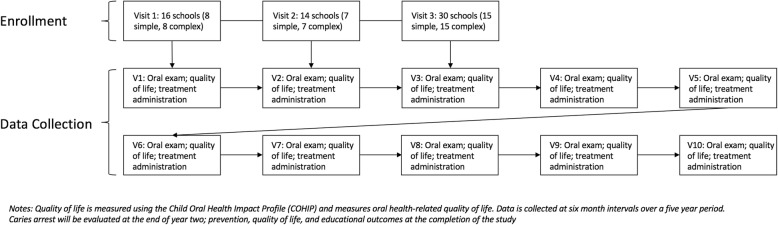
Study design, enrollment, and data collection

Prior to the study, schools meeting the inclusion criteria will be solicited for participation and randomly assigned to receive fluoride varnish/SDF or fluoride varnish/sealants in 6-month intervals (± 1 month). Informed consent for all children in participating schools will be distributed to parents at the beginning of each school year. At each observational period, study participants with completed consent will receive a comprehensive oral examination provided by a licensed dental hygienist and complete a brief psychological assessment for oral health-related quality of life (Fig. 1) [36, 37]. Following the oral evaluation, participants will receive the assigned treatments (Additional file 1).

**Fig. 2:**
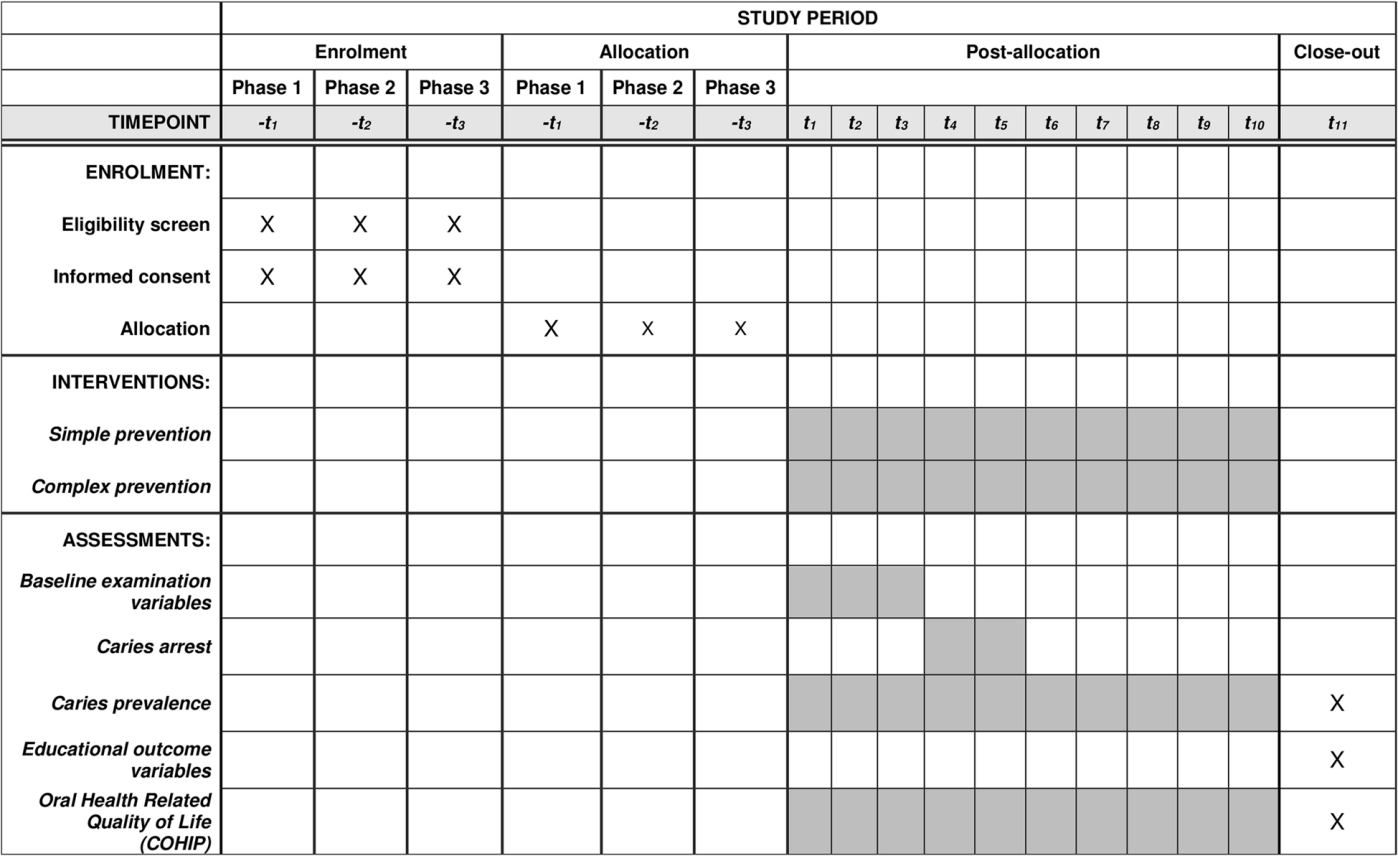
Schedule of enrollment, interventions, and assessments

The primary aim is to evaluate the non-inferiority of SDF versus sealants in the arrest and prevention of dental caries. Secondary study aims include assessing differences in provider type, quality of life, academic performance, and school attendance. To determine provider effects, treatments in the simple prevention group will be given by either dental hygienists or registered dental nurses and the rates of arrest and prevention will be compared across provider. Data for educational outcomes will be solicited from the New York City (NYC) Department of Education (DOE). Any participant presenting with a medical emergency will be referred to school nurses for follow-up care. The anticipated schedule of enrollment, interventions, and assessments is shown in Fig. 2.

### Clinical evaluation and diagnosis

At each observation for each participant, examining dentists will dry tooth surfaces with gauze squares and perform full-mouth oral examinations, including examination of all teeth and tooth surfaces to determine if sound, decayed, missing, filled, or having pulpal involvement. The exam will also include an assessment of pain, swelling, infection, and abscess presence.

Diagnosis of cavitated carious lesions will be made based on a visual-tactile oral examination and follow criteria for cavitated lesions as specified by the *Diagnostic Criteria and Procedures* of the Oral Health Surveys of the National Institute of Dental Research [38]*.*

Examiners will be calibrated by examining ten students independently at baseline and discussing whether caries are present or not. Following this review, examiners will be calibrated by examining another ten students independently and comparing results. To standardized delivery of care, examiners will be trained to use all interventions prior to participating in the study. Standardization of diagnostic criteria will consist of reviewing diagnoses at the mid-point of each study year and discussing with examiners. Both calibration and standardization will be conducted every year of the study.

Treatments in the complex arm will be provided by dental hygienists with the support of dental assistants. Treatments in the simple arm will be provided by registered nurses or dental hygienists with the support of dental assistants, both overseen by a nurse practitioner.

### Treatment description and regimen

#### Simple prevention

One drop (0.05 ml) of SDF (Advantage Arrest™) solution at 38% concentration (2.24 F-ion mg/dose) will be dispensed per child. Posterior tooth surfaces to be treated will be dried, after which the SDF will be applied with a microbrush to all asymptomatic cavitated lesions and to all pits and fissures on bicuspids and molar teeth for 30 s. Fluoride varnish (5% NaF, Colgate PreviDent™) will then be applied to all teeth. Simple prevention will be provided by either dental hygienists or registered nurses.

#### Complex prevention

All primary and permanent teeth will be dried prior to application. Pits and fissures on all bicuspids and molar teeth will be sealed with glass ionomer sealants (GC Fuji IX). Interim therapeutic restorations will be placed on all frank asymptomatic cavitated lesions. Cavitated lesions will be cleaned using a tooth brush prior to placement of ITR. Fluoride varnish (5% NaF, Colgate PreviDent™) will then be applied to all teeth. Complex prevention will be provided by dental hygienists.

Across both arms, non-cavitated lesions will be treated with fluoride varnish. Both arms will also receive toothbrushes, fluoride toothpaste, and oral hygiene instruction. Clinical care will be provided in a dedicated room in each school using mobile equipment and disposable supplies. Any child with pulpal involvement or in need of an extraction will be referred to local dentists for follow-up care.

### Risks and adverse events

Each intervention used in this trial is currently used in clinical practice as a standard of care procedure. The potential risks for study participants are minimal and identical to the risk for children obtaining care in a dental office. The greatest risk is an allergic reaction to fluoride varnish, SDF, or glass ionomer. All adverse events occurring during the study period will be recorded: at each contact with the study participant, investigators will seek information on adverse events by specific questions and an oral examination. Evidence of adverse events will be recorded on electronic health records and appropriate case report forms. The clinical course of each event will be followed until resolution, stabilization, or until it has been determined that participation in the study was not the cause. Serious adverse events ongoing at study end will be followed to determine the final outcome. Adverse event reports will be reported to the IRB within five working days from the time that investigators become aware of the event.

### Definition of outcome measures

#### Primary outcome measures

Primary outcomes include clinically evaluated caries arrest and the prevention of new caries. Caries arrest will be evaluated after 2 years and the prevalence of new caries will be evaluated after 4 years.

#### Secondary outcome measures

Secondary outcomes include oral health-related quality of life measured using the Child Oral Health Impact Profile – Short Form (COHIP-SF) [37], academic performance measured using mathematics and English language standardized assessments given to all NYC school children between grades 3 and 8, and the number of school days missed for each participant over each academic year.

### Recruitment and eligibility

All schools meeting the inclusion criteria were solicited by the NYC DOE to participate in the program. School principals were mailed letters describing the study protocol and interventions, and any interested principal opted into the study. Prior to the beginning of each school year, electronic rosters for each participating school will be provided to study investigators from the DOE, which will include a unique student identifier, contact information, demographic and socioeconomic variables, and Medicaid identification (if available). School rosters will be used to electronically create personalized informed consent for every student in the school, which will then be combined with a letter from the principal explaining the study and distributed to parents of children in each school. Completed informed consent will be collected at the school by NYU investigators. Schools will be recruited and enrolled over the first 2 years of the study. However, children within schools will be consented and enrolled in each year of the program to accommodate newly registered students each academic year. Recruitment for this study is pending.

#### Inclusion criteria

Any primary school in NYC with a student Hispanic/Latino population greater than 50% and a low-income population (defined as a student receiving free or reduced-price lunch) of at least 80% is eligible to participate. Within participating schools, all children are eligible to participate in the study regardless of age, sex, race/ethnicity, insurance status, or other sociodemographic variable. Those with informed consent and assent will receive care.

#### Exclusion criteria

The only schools that are ineligible to participate are those that already have a pre-existing school-based dental health program. Within participating schools, exclusion criteria for children include those without informed consent or those with consent but without assent.

### Randomization

Participating schools will be block randomized at the school level to receive either the simple or complex treatment using a random-number generator. First, schools will be ordered by the total student population. Blocks of two schools will then be selected sequentially from the list. Schools in each block will then be randomly assigned to either simple or complex arms using a random-number sequence generated from a computer program. Schools will enter into the study in three phases: in phase 1, 16 schools will be randomized. Six months later (phase 2), an additional 14 schools with be randomized, followed by 30 more schools 6 months after that (phase 3).

Block randomization will be conducted at the school level as the NYC Departments of Education and Health and Mental Hygiene requested that interventions be identical within schools. Additionally, interventions in the simple arm are provided by nurses and hygienists, while treatments in the complex arm are provided by hygienists only. Thus, study coordination is more feasible if provided at the school level.

### Blinding

Due to the nature of the treatments, nurses and dental hygienists providing care will not be independent from study protocols and, therefore, are not blinded. Additionally, all participants will be provided with a letter describing the care that they received. Assignment to treatments will follow a predetermined randomization list at the school level, and all students with consent in participating schools will receive the assigned treatment. However, all data for caries arrest will be masked prior to analysis such that which schools were assigned to which treatment, and the subsequent treatments given to each participants (e.g., SDF or ITR), will not be able to be determined during analysis. Only data on the sound or decayed/filled status of individual teeth will be provided per participant. Following analysis of caries arrest, this can no longer be guaranteed.

### Data collection, transmission, and storage

Data collected from each participant will be recorded on a password-protected tablet computer using a propriety software system that is pre-populated with the demographic information of the participant. Recorded data will also include responses to the short form version of the Child Oral Health Impact Profile (COHIP-SF). COHIP questions will be posed to each participant and examiners will record responses.

Following each day of the study, electronic records will be uploaded to a secure server and stored at the Boston University Data Coordinating Center (DCC) and evaluated for quality assurance. The DCC will also maintain sociodemographic information provided by the NYC DOE for each study participant. When the DCC transmits data to investigators, no identifying information will be provided with the exception of a unique student identifier. This data will be kept at the NYU on a secure, password-protected server.

Data for secondary school outcomes (academic performance and attendance) will be transmitted to the NYU investigators by the NYC DOE at the end of each school year.

### Sample size calculation

The presented study is powered for the primary outcomes of caries arrest and prevention. Approximately 14,100 students are expected to be enrolled across 60 schools over the duration of the study (*n* = 235 per school cluster). Based on a pilot study of school-based caries prevention conducted in NYC from 2016 to 2017, the baseline prevalence of caries of the primary and permanent dentition at the child level was 40.7%. The prevalence of untreated decay on primary dentition only was 36.4%, and the prevalence of untreated decay on permanent dentition only was 35.1%. The average dmft was 1.40 (SD = 2.04) and the average DMFT was 0.34 (SD = 0.84). Power for the primary outcome of caries arrest was calculated using a two-arm non-inferiority trial design. The per-person proportion of carious teeth at baseline that were treated with complex or simple prevention and stayed arrested over a 2-year period will be analyzed at the child level. Calculations assume an equal proportion of success, *π*, of teeth with caries being arrested in complex and simple arms. The non-inferiority margin (*δ*) was set at 10%. Additional parameters of a two-sided type-I error rate of 5% and statistical power of 0.80 yielded a total sample size per group of 198 (*n*_tot_ = 396) [39]. To adjust for any potential school-level clustering based on inclusion criteria of schools consisting of high-need children, an intraclass correlation coefficient (ICC) of 0.10 was assumed. The design effect associated with this ICC increased the effective sample size by a factor of 24.5, resulting in a total required sample of 9702.

For caries prevention, power for the two-arm, longitudinal, cluster randomized design was estimated using the method of Diggle et al. (2002) for generalized estimating equations [40]. Estimates assume an average number of visits per child of six, statistical power of 0.80, and a two-sided type I error rate of 5%. A repeated measures correlation of 0.5 and a per-visit attrition rate of 20% were also assumed. For a given minimally detectable effect size (standardized effect size difference) of 0.25, a cluster-adjusted (ICC = 0.10) sample size of 12,874 is required. Based on the expected participant enrollment, the study is powered for these conservative assumptions for caries prevention.

For secondary outcomes, the NYC citywide average absenteeism rate is 14.2%. The average performances for reading and math examinations (grades 3–5) are 298 (SD = 17) and 299 (SD = 21), respectively. Sample sizes were calculated using a simple two-group cluster randomized comparison of means (standardized test performance) and proportions (school absences). Power calculations assume an intraclass correlation of 10%, statistical power of 0.80, and a two-sided type I error rate of 5%. Based on these assumptions, the anticipated sample size is sufficient to detect a 9% decrease in absenteeism (to 5%) and a standardized test difference of 6.44 (reading) and 7.95 (math).

Quality of life will be measured using the short form version of the Child Oral Health Impact Profile (COHIP-SF) [37]. In a validation study of the COHIP-SF, a pediatric sample of children (aged 7–17 years) had average scores of 56.2 (SD = 9.3). For a simple cluster randomized, two-group mean comparison, with a two-sided type I error rate of 5%, statistical power of 0.80, and ICC of 0.10, the study is powered to detect a difference of 2.2 on the COHIP-SF scale.

### Statistical analysis

For the non-inferiority of caries arrest, the per-patient proportion of carious lesions at baseline treated with simple versus complex prevention that stayed arrested throughout the first 2 years of study observation will be determined. Any deciduous teeth with treated carious lesions that are lost due to exfoliation during the course of the study will be considered as arrested throughout the observed lifetime of the tooth. Data will record the last observed status of exfoliated teeth to be used in subsequent analyses. Thus, tooth-level indicators are able to be present for both primary and permanent dentitions at the same time. With this approach, each carious tooth treated with either simple or comprehensive prevention is a single trial with outcomes either of arrested (1) or failed to arrest (0). The percentage of arrested caries (at the child level) will be modeled using multilevel binomial regression with a logit link:

$$ {Y}_j\sim B\left({\pi}_{\mathrm{j}}\right),E\left({Y}_{\mathrm{j}}\right)={\pi}_{\mathrm{j},} $$

where *π*_j_ is the probability of success (as defined above). Analysis will be conducted for all teeth, permanent teeth only, and primary teeth only. As described, the non-inferiority margin, *δ*, is set at 10%. While there is no “gold standard” criterion for the selection of this margin, the margin was set based on collaborative discussion with clinicians to determine what is considered clinically unimportant. The null hypothesis is that the experimental treatment (simple prevention) is inferior to the standard treatment (complex prevention) by at least *δ*: *π*_*simple*_ *− π*_*complex*_ ≥ *δ*. The alternative hypothesis is that *π*_*simple*_ − *π*_*complex*_ < *δ*.

Based on results from multilevel binomial models, differences in effect sizes estimated by confidence intervals will be used to determine clinical non-inferiority of the two prevention methods [41]. Confidence intervals will be calculated for the difference between the two interventions, with the width of this interval signifying the extent of non-inferiority. If the difference between the two interventions lies to the right of *δ*, then non-inferiority will be concluded. Though this is method is preferred by reporting guidelines, *p* values will also be reported, in keeping with other recommendations [41].

For the prevention of new caries, longitudinal data will be analyzed using generalized estimating equations (GEE) and mixed-effects multilevel regression models (ME-MLM) with the appropriate error distribution for the prevalence and incidence of untreated caries. The number of teeth at risk for each child during each follow-up interval will be identified and the number of those teeth in which new caries is observed at the examination that ends that interval will be determined. Primary teeth lost in each interval and new permanent teeth will not contribute to data for that interval. Data from baseline visits will be omitted from analyses and used as an indicator of any untreated decay at baseline. Analysis will be conducted for all teeth, permanent teeth only, and primary teeth only.

To explore non-linear trends in untreated decay between simple and complex prevention, longitudinal data will be analyzed using generalized additive models (GAMs) with non-parametric smoothers, linking the known proportion:

$$ {p}_{it}=\mathrm{E}\left({\mathrm{y}}_{\mathrm{it}}=1|{\mathrm{x}}_{\mathrm{ijt}},{\mathrm{z}}_{\mathrm{it}}\right) $$

to a non-linear non-parametric predictor using the link function:

$$ {n}_{it}=g\left({u}_{it}\right)=\ln \left({u}_{it}/1-{u}_{it}\right)={\sum}_{j=1}^p{s}_j\left({x}_{jit}\right)+{z}_{it}^T{u}_i, $$

where *s*_j_ are smooth non-parametric functions and *u*_i_ are random effects assumed to be iid ~ *N*(0, D(ψ)) [42]. Heterogeneity and correlation among subjects will be accounted for through random effects.

Longitudinal effects of simple and complex prevention on academic outcomes, compared to untreated children, will be analyzed using propensity score matching and multilevel modeling. First, propensity scores will be estimated for each participant at baseline for the probability of treatment assignment conditional on observed covariates (e.g., prior academic performance). Propensity scores will be used to match treatment students to students not receiving treatment, considering multiple forms of matching such as nearest neighbor and caliper. Potential comparator students not receiving either simple or complex treatments will be drawn from “peer-schools,” schools identified by the NYC DOE as similar to treated schools based on socioeconomic, academic performance, and teacher-quality indicators. This data is anonymized and can be used without consent as a secondary data source. Treated students and matched comparators will then be analyzed using ME-MLM (for academic achievement) and Poisson regression (for school absences). If a different mechanism drives initial versus continued absences, school absences will be analyzed using zero-inflated negative binomial multilevel modeling.

Finally, change in OHRQoL between groups over time will be analyzed using ME-MLM. Baseline quality of life will be included as a covariate and models will include predictors for time, treatment, and the treatment-time interaction. Hypothesized a priori confounders, including sociodemographic variables at the child and school level, will be included.

Missing data will be adjusted for using multiple imputation and inverse probability weighting (IPW). Statistical analysis will be performed following intention-to-treat and analyzed using Stata v14.0 (StataCorp LLC, College Station, TX, USA) and R v3.1.1.

## Discussion

Due to the considerable oral health needs of high-risk children resulting from access barriers to traditional office-based care, school-based caries prevention programs have become a popular public health dentistry intervention [43]. The vast majority of these programs use dental sealants as the primary preventive agent, though others include fluoride varnish and ITR [44, 45]. Despite a growing interest in SDF to arrest and prevent caries in clinical trials [46, 47], to our knowledge they have never been used in school-based prevention programs in the United States. Given the economic benefits of SDF and because they can be provided to patients much faster than traditional sealants, demonstrating the non-inferiority of SDF compared to traditional sealants in the arrest and prevention of caries has the potential to drastically alter the landscape of school-based oral health care.

Research has shown that tooth decay is responsible for negative effects on oral health-related quality of life [10, 20] as reported by both children who experienced decay and their caregivers [48], and is also associated with functional limitations as measured by different OHRQoL scales [49]. Furthermore, poor oral health can directly lower school performance in children [50, 51] and result in increased absenteeism [52], either directly through oral pain or through reduced quality of life [53]. For example, one study has shown that children with toothache are four times as likely to have a low grade-point average, and dental health issues were responsible for 35% of a child’s missed school days [18]. This suggests that improving oral health can directly impact educational outcomes. The presented clinical trial will thus additionally evaluate differences between caries prevention agents on key domains of quality of life, particularly oral health, functional well-being, and socio-emotional well-being, as well as academic performance and school attendance.

This study will be conducted in parallel with a companion trial of SDF versus traditional sealants in schools in New Hampshire, with notable differences [54]. The primary study population for the presented trial is low-income urban Hispanic/Latino children with access to fluoridated water, though enrollment is open to all children regardless of race/ethnicity and socioeconomic status. In contrast, the companion study will recruit primarily low-income white rural children without access to water fluoridation. Additionally, this latter trial will also determine the cost-effectiveness of simple versus complex prevention, while the former includes oral health-related quality of life, academic performance, and school attendance outcomes. Finally, the presented study will include a comparison of the effectiveness of SDF when applied by school nurses versus dental hygienists. Following primary analyses, the proportion of caries arrested and the rate of new caries observed will be compared between children who received simple prevention from a school nurse and children who received simple prevention from a dental hygienist. Importantly, the simplicity of applying SDF means that if similarly effective, the existing registered nurse workforce present in schools can easily provide preventive oral health care.

The direct benefit anticipated for participating children includes improved oral health, positive increases in quality of life, and improved school attendance and performance. Due to the minimally invasive nature of experimental interventions, no additional risks are expected. This study could lead to broader utilization, provided by existing school nurses, of SDF in schools.

### Trial status

Protocol version 1.0 (11/10/17). Recruitment will begin in September 2018. Recruitment will be on a rolling, semester-by-semester basis and will conclude in June 2022. The trial is registered with ClinicalTrials.gov (22 February 2018; NCT03442309; available URL: https://clinicaltrials.gov/show/NCT03442309.

## Additional file

Additional file 1: Standard Protocol Items: Recommendations for Interventional Trials (SPIRIT) 2013 Checklist: recommended items to address in a clinical trial protocol and related documents*. (DOC 122 kb)

## Abbreviations

COHIP-SF: Child Oral Health Impact Profile – Short Form
DCC: Data Coordinating Center
DOE: Department of Education
GEE: Generalized estimating equations
IPW: Inverse probability weighting
ITR: Interim therapeutic restorations
ME-MLM: Mixed-effects multilevel models
NYC: New York City
NYU: New York University
SDF: Silver diamine fluoride
SPIRIT: Standard Protocol Items: Recommendations for Intervention Trials

## References

[1] Kidd E (2011). The implications of the new paradigm of dental caries. J Dent.

[2] Marcenes W (2013). Global burden of oral conditions in 1990-2010: a systematic analysis. J Dent Res.

[3] Kassebaum NJ (2017). Global, regional, and national prevalence, incidence, and disability-adjusted life years for oral conditions for 195 countries, 1990–2015: a systematic analysis for the global burden of diseases, injuries, and risk factors. J Dent Res.

[4] Dye B, Li X, Thornton-Evans G (2012). Oral health disparities as determined by selected healthy people 2020 oral health objectives for the United States, 2009–2010. NCHS Data Brief.

[5] Dye BA, et al. Dental caries and sealant prevalence in children and adolescents in the United States, 2011–2012. NCHS Data Brief. 2015;(191):1–8. 25932891

[6] Griffin SO (2016). Vital signs: dental sealant use and untreated tooth decay among U.S. school-aged children. MMWR Morb Mortal Wkly Rep.

[7] Meyer F, Enax J (2018). Early childhood caries: epidemiology, aetiology, and prevention. Int J Dent.

[8] Ferraz NK (2014). Clinical consequences of untreated dental caries and toothache in preschool children. Pediatr Dent.

[9] Frencken JE (2017). Global epidemiology of dental caries and severe periodontitis—a comprehensive review. J Clin Periodontol.

[10] Onoriobe U (2014). Effects of enamel fluorosis and dental caries on quality of life. J Dent Res.

[11] Ramos-Jorge J (2014). Impact of untreated dental caries on quality of life of preschool children: different stages and activity. Community Dent Oral Epidemiol.

[12] Krisdapong S, Somkotra T, Kueakulpipat W (2014). Disparities in early childhood caries and its impact on oral health-related quality of life of preschool children. Asia Pac J Public Health.

[13] Farber J. Oral health and the commonwealth’s most vulnerable children: a state of decay. Boston: The Massachusetts Society for the Prevention of Cruelty to Children (MSPCC); 2004.

[14] Blumenshine SL (2008). Children’s school performance: impact of general and oral health. J Public Health Dent.

[15] Seirawan Hazem, Faust Sharon, Mulligan Roseann (2012). The Impact of Oral Health on the Academic Performance of Disadvantaged Children. American Journal of Public Health.

[16] Detty Amber M.R., Oza-Frank Reena (2014). Oral health status and academic performance among Ohio third-graders, 2009-2010. Journal of Public Health Dentistry.

[17] Paula JSD, Mialhe FL (2013). Impact of oral health conditions on school performance and lost school days by children and adolescents: what are the actual pieces of evidence?. Brazilian Journal of Oral Sciences.

[18] Jackson SL (2011). Impact of poor oral health on children’s school attendance and performance. Am J Public Health.

[19] Pourat N, Nicholson G. Unaffordable dental care is linked to frequent school absences. Los Angeles: UCLA Health Policy Research Brief: UCLA Center for Health Policy Research; 2009. 19960616

[20] de Paula JS (2015). Longitudinal evaluation of the impact of dental caries treatment on oral health-related quality of life among schoolchildren. Eur J Oral Sci.

[21] Treadwell HM (2017). The nation’s oral health inequities: who cares?. Am J Public Health.

[22] Dye BA, Thornton-Evans G. (2010). Trends in oral health by poverty status as measured by Healthy People 2010 objectives. Public Health Rep (Washington, D.C. : 1974).

[23] Polk DE, Weyant RJ, Shah NH, Fellows JL, Pihlstrom DJ, Frantsve-Hawley J. Barriers to sealant guideline implementation within a multi-site managed care dental practice. BMC Oral Health. 2018;18(1):17. 10.1186/s12903-018-0480-z PMC5797385 29394921

[24] P.H.S. U.S. Department of Health and Human Services, Centers for Disease Control and Prevention, and the National Institutes of Health, National Institute of Dental and Craniofacial Research (2003). A national call to action to promote oral health.

[25] Griffin SO (2014). Use of dental care and effective preventive services in preventing tooth decay among U.S. Children and adolescents—Medical Expenditure Panel Survey, United States, 2003–2009 and National Health and Nutrition Examination Survey, United States, 2005–2010. MMWR Surveill Summ.

[26] Dye BA (2007). Trends in oral health status: United States, 1988–1994 and 1999–2004. Vital Health Stat 11.

[27] Ruff RR. Total observed caries experience: Assessing the effectiveness of community-based caries prevention. J Public Health Dent. 2018. 10.1111/jphd.12284. 10.1111/jphd.12284 PMC6279466 30114726

[28] U.S.G.A. Office (2010). Oral Health: efforts under way to improve children’s access to dental services, but sustained attention needed to address ongoing concerns.

[29] Marinho VC (2013). Fluoride varnishes for preventing dental caries in children and adolescents. Cochrane Database Syst Rev.

[30] Ahovuo-Saloranta A (2013). Sealants for preventing dental decay in the permanent teeth. Cochrane Database Syst Rev.

[31] Yengopal V (2009). Caries-preventive effect of glass ionomer and resin-based fissure sealants on permanent teeth: a meta analysis. J Oral Sci.

[32] de Amorim RG, Leal SC, Frencken JE. Survival of atraumatic restorative treatment (ART) sealants and restorations: a meta-analysis*.* Clin Oral Investig, 2012. 16(2): p. 429–441. 10.1007/s00784-011-0513-3 PMC3308010 21274581

[33] Zhi Qing Hui, Lo Edward Chin Man, Lin Huan Cai (2012). Randomized clinical trial on effectiveness of silver diamine fluoride and glass ionomer in arresting dentine caries in preschool children. Journal of Dentistry.

[34] Rosenblatt A., Stamford T.C.M., Niederman R. (2009). Silver Diamine Fluoride: A Caries “Silver-Fluoride Bullet”. Journal of Dental Research.

[35] IOM (2009). Initial National Priorities for Comparative Effectiveness Research.

[36] Broder HL, Wilson-Genderson M (2007). Reliability and convergent and discriminant validity of the Child Oral Health Impact Profile (COHIP Child’s version). Community Dent Oral Epidemiol.

[37] Broder Hillary L., Wilson-Genderson Maureen, Sischo Lacey (2012). Reliability and validity testing for the Child Oral Health Impact Profile-Reduced (COHIP-SF 19). Journal of Public Health Dentistry.

[38] U.S.D.o.f.a.H. Services (1991). Oral health surveys of the National Insitute of Dental Research: Diagnostic criteria and procedures.

[39] Chow S, Shao J, Wang H. Sample size calculations in clinical research. CRC Biostatistics Series, ed. C. Hall. 2008, Boca Raton: Taylor & Francis.

[40] Diggle PJ (2002). Analysis of longitudinal data.

[41] Greene CJ (2008). Noninferiority and equivalence designs: issues and implications for mental health research. J Trauma Stress.

[42] Sapra S (2014). Semi-parametric mixed effects models for longitudinal data with applications in business and economics. International Journal of Advanced Statistics and Probability.

[43] Gooch BF (2009). Preventing dental caries through school-based sealant programs: updated recommendations and reviews of evidence. J Am Dent Assoc.

[44] Ruff RR, Niederman R (2018). Comparative effectiveness of school-based caries prevention: a prospective cohort study. BMC Oral Health.

[45] Gooch BF (2009). Preventing dental caries through school-based sealant programs. J Am Dent Assoc.

[46] Llodra JC (2005). Efficacy of silver diamine fluoride for caries reduction in primary teeth and first permanent molars of schoolchildren: 36-month clinical trial. J Dent Res.

[47] Horst JA, Ellenikiotis H, Milgrom PM. UCSF protocol for caries arrest using silver diamine fluoride: rationale, indications, and consent. J Calif Dent Assoc. 2016;44(1):16–28. PMC4778976 26897901

[48] Abanto J (2014). Impact of dental caries and trauma on quality of life among 5- to 6-year-old children: perceptions of parents and children. Community Dent Oral Epidemiol.

[49] Mota-Veloso I (2016). Impact of untreated dental caries and its clinical consequences on the oral health-related quality of life of schoolchildren aged 8-10 years. Qual Life Res.

[50] Paula JS (2016). School performance and oral health conditions: analysis of the impact mediated by socio-economic factors. Int J Paediatr Dent.

[51] Garg N, Anandakrishna L, Chandra P. (2012). Is there an association between oral health status and school performance? A preliminary study. Int J Clin Pediatr Dent.

[52] Krisdapong S (2013). School absence due to toothache associated with sociodemographic factors, dental caries status, and oral health-related quality of life in 12- and 15-year-old Thai children. J Public Health Dent.

[53] Piovesan C (2012). Influence of children’s oral health-related quality of life on school performance and school absenteeism. J Public Health Dent.

[54] Ruff RR, Niederman R (2018). Comparative effectiveness of treatments to prevent dental caries given to rural children in school-based settings: protocol for a cluster randomised controlled trial. BMJ Open.

